# Twenty-first Annual Convention of the American Medical Association

**Published:** 1870-05

**Authors:** 


### Miscellaneous.

### Twenty-first Annual Convention of the American Medical Association.

The convention was called to order at 11 A. M., by the president, George Mendenhall, of Ohio; William B. Atkinson, of Philadelphia, secretary.

Professor F. G. Smith, of Pennsylvania, L. A. Sayre, of New York, Warren Stone, of Louisiana, and J. S. Moore, vice presidents, took seats on the stage beside the president. The ex-presidents of the society also took seats on the stage.

The Rev. Dr. Boynton then opened the convention with a fervent prayer.

The President then announced that the report of the Committee of Arrangements would be read.

---

Dr. Antisell, chairman of that committee, then delivered a speech of welcome, expressing himself as gratified in seeing so full a representation.

The reports of the old committees were made and referred to the proper sections.

The committee then presented the following programme:

PROGRAMME FOR EVENINGS OF MAY 3, 4, AND 5.

Tuesday—Reception by the President of the United States at 8 P. M.

Wednesday—Reception by the Surgeon General, at the Army Medical Museum, from 7 to 10 P. M.; surgical lecture in the lower hall at 8 P. M.; microscopical lecture in the lower hall at 8.45 P. M.

Thursday—Exhibition of the illumination of the Capitol dome at 8 P. M.; reception by the Mayor of Washington, Hon. S. J. Bowen, at 9 P. M.

The secretary then read the roll of membership.

The Committee on Credentials submitted a majority report, which excluded delegates from the National Medical Society of the District of Columbia, American Academy of Medicine of the District of Columbia, Howard University Medical College, Alumni Association of the Medical department of Georgetown College; also, the three city hospitals, because they consult with colored and irregular physicians.

Dr. Robert Reyburn, chairman of the Committee on Credentials, submitted a minority report. He began by remarking that the committee had disgraced itself and lowered itself to the level of a political caucus.

Dr. Davis, of Chicago, called the gentleman to order.

The minority report as made by Dr. Reyburn, is as follows:—

The undersigned respectfully protests against the admission to the approaching session of the American Medical Association of the delegates from the Medical Society of the District of Columbia for the following reasons, viz.:

These delegates represent a society which, in open defiance of the ethics of the American Medical Association, for the fee of ten dollars, issues licenses to practice medicine in the District of Columbia to homœopathic and other irregular practitioners.

This society is also irregular, and violates the ethics of the American Medical Association by claiming and exercising the power to grant licences to practice medicine in the District of Columbia, to persons who are not graduates of any respectable medical college, for the fee of ten dollars.

The undersigned also respectfully protests against the admission to the next session of the American Medical Association, of the delegates from the so-called Medical Association of the District of Columbia, for the reason that said association is composed of the same individuals that form the Medical Society of the District of Columbia, in fact, it only settles the fee bill and local ethics of the medical profession of the District, and can in no sense be called a medical organization entitled to representation in the American Medical Association.

No medical papers, essays, or pathological specimens are printed at its meetings, and it is in fact only an ingenious device by which the Medical Society of the District of Columbia is enabled to duplicate its number of delegates in the American Medical Association.

The undersigned also respectfully calls attention to the number of delegates claiming to represent the medical profession of the District of Columbia. The total number of regular physicians in the District is about two hundred, which would give about twenty delegates, and yet it will be seen that the District delegates number about sixty-four, which is evidently unfair, and gives the District a much larger representation than it is justly entitled to.

The undersigned having already filed a written protest with the Committee on Credentials, for the reasons above given, respectfully recommends that certain gentlemen, delegates from the Medical Society of this city, be refused admission to the approaching session of the association.

The undersigned reports favorably upon the credentials of, and recommends that seats be granted to, certain named gentlemen, delegates excluded by the majority report, representing various societies and medical institutions of the District.

On motion, the report was accepted and referred to the Committee on Ethics, with instructions to report at their earliest convenience.

Dr. Tucker, of California, then moved that so much of the majority report as affected the minority report be referred to the Committee on Ethics.

Dr. Stewart, of the District of Columbia, presented a protest to the majority report of the Committee on Credentials; which was also referred to the Committee on Ethics.

Dr. Busey, of the District of Columbia, presented a protest signed by many physicians against the admission of Dr. C. C. Cox, of Maryland, as a representative from that State; which was referred to the Committee on Ethics.

Dr. Davis, of Illinois, said if they at this stage of the session gave members the right of discussion on non-important points that there would be no time for business. He hoped, therefore, that no discussion would be allowed until the regular committees had reported.

Dr. Martin, of Massachusetts, said that as a majority of the delegates of Massachusetts had been excluded for some unexplained cause, he therefore moved that the subject be referred to the Committee on Ethics. It was so referred.

Dr. Davis moved that all questions pertaining to the right of institutions, hospitals, colleges, and private persons, as to their admission into the convention, be all referred to the Committee on Ethics. The motion prevailed.

Dr. Davis then moved that the meeting proceed with the regular order of business. Carried.

A number of members were then accepted by invitation.

The reading of letters and telegrams from absent members was next in order.

Dr. Lewis A. Sayre, chairman of the Committee on Ethics, and one of the vice presidents of the association, said a pamphlet had been published and circulated by one Dr. Ruppaner, and asked that the subject be referred to a special committee.

Dr. Davis moved that the Chair appoint a new Committee on Ethics for the ensuing year, to which this subject might be referred. Carried.

The following gentlemen were appointed: Alfred Stille, New York; N. S. Davis, Illinois; J. N. Keller, Kentucky; H. P. Askew, Delaware; J. J. Woodward, U. S. Army.

The convention then took a recess of five minutes.

After the reassembling of the convention, the President read his annual address.

It was moved that the thanks of the association were due to the President for his able address, and that a copy be requested for publication. Carried.

The next business was the report of special committees and presentation of papers.

The committees for the year 1869 made their reports, and some of which were continued and others discharged.

Dr. T. Antisell, District of Columbia, then read a report on veterinary colleges; which was referred to the Committee on Printing.

Dr. C. C. Cox, of Maryland, moved that the name of Dr. Busey, of the District of Columbia, be stricken out of the list of delegates until such time as the Committee on Ethics should report relative to the District of Columbia.

Dr. Busey said that Dr. Cox was not a delegate from Maryland.

A vote was taken on the motion of Dr. Cox. It was lost.

There was much discussion on this subject, when it was moved that the delegates of the District of Columbia find a room in which to fight out their battles. [Laughter.]

The Secretary then announced that the Nominating Committee would meet in the prayer-room at 4 P. M.

### SECOND DAY'S SESSION.

The Convention assembled at 10 o'clock. Prof. George Mendenhall, president, in the chair, and Dr. William B. Atkinson, secretary.

Dr. Gross, of Pennsylvania, offered a resolution that a social reunion be held annually on the third day of the session. He moved that a supper be had at the Arlington to-morrow evening at 8 o'clock. Carried.

A committee of five was appointed by the Chair to carry out the objects of the above.

A number of members were admitted by invitation.

Dr. A. Stille, of Philadelphia, made a partial report in relation to the admission of the Massachusetts delegation to seats in the convention, in favor of admitting all those that have been registered. The report was accepted.

It was moved that the vote referring the paper of Dr. Antisell on veterinary colleges, to the publication committee, be reconsidered. It was so ordered, and the paper was referred to a special committee of three.

A protest was read on the admission of Dr. C. C. Cox, of Maryland, charging him with a violation of the code of ethics, and practising medicine without license. Referred to the Committee on Ethics.

Dr. Cox was permitted to explain, saying that he had paid $10 for membership in the Medical College of Washington, and commenced the practice of medicine in this city in July last, but to this day he had not received his license from that society.

Dr. Palmer, of the District of Columbia, arose to reply, but privilege was not given, as the whole subject had been referred to the Committee on Ethics.

Dr. W. H. Murray, of Ohio, moved that the Committee on Ethics retire immediately and prepare their report, and sit until they had come to some final settlement in regard to the delegates not declared members by the Committee on Credentials. The motion was not agreed to.

Dr. Loomis, of the District of Columbia, moved that all delegates from the District of Columbia be permitted to occupy seats in the convention until the right to their seats has been decided by the Committee on Ethics.

The motion was put and decided in the negative by a vote of 107 yeas to 152 nays.

Dr. C. C. Cox moved that "*none*" of the delegates from the District of Columbia be admitted until the Committee on Ethics report. Not agreed to.

Dr. D. W. Yandell, of Kentucky, moved that the District of Columbia be blotted out from the map of the American Medical Association. Laid on the table.

Dr. F. G. Smith, of Pa., of the Committee on Publication, made a report of the work of the committee during the past year. The report was received and referred.

Dr. Gross offered a resolution providing for the publication of the proceedings of the association in some medical journal published monthly. Adopted.

After some debate the vote was reconsidered on the adoption of the above, and the subject referred to a special committee.

The annual report of the treasurer was then submitted, received, and referred to the Committee on Publication.

Dr. Gross, of Pa., presented a letter from the British Medical Association, cordially approving of the objects of this Association. Referred.

Dr. T. A. Antisell rose to a privileged question, stating that the publication in the *Morning Chronicle*, alluding to the Committee of Arrangements, asserting that the committee had insulted prominent city physicians, he stigmatized as false, and said that the article in "proof-slips" was strewn broadcast throughout the hall for political purposes.

On motion, it was ordered that no more advertisements, pamphlets, &c., be distributed in the hall during the session of the association, without special permission.

Dr. Robert Reyburn, of D. C., chairman of the Committee on the Library, submitted the annual report of the librarian, which was referred to Committee on Publications.

Prof. L. Sayre, of N. Y., moved that the papers in the hands of the Committee of Ethics, of last year, be referred to the new committee, and that the old committee be discharged. Agreed to.

Dr. T. Antisell, of the Committee of Arrangements, read a long list of new members who had arrived in the city since the opening of the convention.

Dr. J. J. Woodward, U. S. A., chairman of the Committee on Medical Literature, made a report, which was referred to the Committee on Publication.

Dr. Buck, of New York, moved that the vote by which the resolution providing for a supper at the Arlington on Thursday night be reconsidered, as an invitation had been accepted by the association to call on Mayor Bowen on that evening, and it would be regarded as an insult by the chief executive of the city were the association to fail to call on that functionary as prearranged.

The vote was reconsidered, and the resolution laid on the table.

Dr. C. C. Cox, of Md., chairman, made a report, which was not read, on the subject of American Medical Necrology, and it was referred to the Committee on Publication.

Dr. Moore, of Mo., offered a resolution providing for a uniform price for a course of medical lectures by all members of the profession, and that colleges might charge a higher rate, but not below a certain figure. By this means competition between the schools would elevate the standard of the profession.

Dr. W. G. Davis, of Illinois, said he would be glad to see the day when there would be some uniform proximity to fee, but he did not think it possible for the association to fix an absolute fee that would be binding on the medical colleges of the country. He did not oppose the resolution if the association would name what should be the standard of collegiate medical education.

Dr. Moore said he only desired to fix a minimum fee by his resolution.

Dr. Seldon, of Ohio, moved to fill the blank in the resolution with the sum of $100.

Dr. Somerville, of Mass., seconded the motion.

The blank was filled; and the resolution being before the house,

Dr. Yandell spoke against its passage, saying that it was contrary to the spirit of the association, and contrary to the spirit of the age, to adopt a specific fee for instruction. Our spirit is to educate without reference to compensation. He thought it was competent for institutions to teach for $50, or any other sum it might elect. He ignored the Latin and the Greek. but went in for the English, and he knew of many Latin and Greek scholars who could not write or speak good English. Without there was State legislation on the subject it were folly for this association to attempt to adopt a uniform price for education ; schools regulate themselves. The schools throughout the country maintain themselves, and yet some charge only $50, while others get $150, and the instruction in one is of no higher degree of excellence than the other. The great law of supply and demand goes on ; education, like all other commodities, must regulate itself. Let us charge $1,000 if we can get it, and $50 if we cannot get more.

After further discussion, it was moved and seconded that the resolution be laid on the table. The vote was taken. and the resolution was laid on the table.

Dr. Sullivan, of Mass., moved the action of the association to-day be final for five years.

Dr. Johnson, of Mo., said he was happy to be able to gain the floor, and revived the question of stated fees, arguing that the association had the authority to control the subject of medical education in this country. He implored the body to act, and not indorse any such action as indorsing the proceedings of to-day for five years.

Dr. Sullivan said his reason for making the motion was that the subject of money would not come up again, yet he agreed with the previous speaker that the association had authority to regulate such matters.

Dr. Hubbard, of Connecticut, regarded the motion as out of order, as it would change the organic law of this body if it prevailed.

The motion of Dr. Sullivan was laid on the table.

Dr. Collins, of Massachusetts, offered a resolution recommending that the least sum charged for medical examination for life insurance companies be $5 for each examination. Adopted.

Dr. Horner, of Virginia, introduced a resolution regulating fees of physicians, which was laid on the table.

Dr. Sullivan offered a resolution that the association have power to control medical education throughout the United States. Passed.

Dr. D. O. Donnely, of Maryland, offered a resolution providing for the appointment of a committee of three to report at the next annual meeting such measures as will tend to discountenance the practice of abortionists, and report the names of any belonging to this association, who may be guilty of the practice. Passed.

The committee on permanent organization reported the following officers for the ensuing year: President, Alfred Stille, Pa. ; vice-presidents, J. S. Wetherly, Ala. ; Henry Gibbons, Cal. ; J. T. Hurd, Texas ; Samuel Willey, Minnesota · assistant secretary, Dr.

J. C. Tucker, Cal.; treasurer, Casper Wister. Pa.; librarian, Dr. F. A. Ashford, D. C.

It was resolved that the next place of meeting of the association be at San Francisco, Cal., on the first Tuesday in May, 1871.

The secretary presented a number of communications relating to various subjects, which were referred to committees to be acted on at the next annual meeting.

Dr. Maddox, of Baltimore, asked for information concerning the delegates from the District of Columbia, as to their right to seats in this body.

The Chair informed him that all the delegates of the District had been excluded from seats in the association.

Dr. M. then moved a reconsideration of the vote by which they had been excluded, but he was ruled out of order.

Several amendments to the constitution were made looking to a more efficient working of the association.

A resolution was offered which excluded all delegates who have received their appointments from permanent medical colleges, hospitals, lunatic asylums, and from the American Association at Paris, and that those only should be admitted who received their apppointment from some society in good standing.

The resolution was discussed at length and laid on the table.

Dr. Maddox, of Baltimore, gave notice that unless the Committee on Ethics reported to-morrow morning why the regular recognized physicians of this city were excluded from seats in this association he would move a reconsideration of the vote which excluded them, and move for their admission.

A partial report from the majority of the Committee on Ethics was submitted, wherein it was cited, that nothing in the constitution prohibited medical societies in good standing from sending delegates to the association. The committee reported in favor of the admission of Dr. C. C. Cox, of Maryland, to a seat in this body.

A minority report was also submitted, reciting that delegates to the association should be permanent residents of the place where they practice their profession, and therefore Dr. Cox is not entitled to a seat.

The reports were received, and, on motion, the majority report was adopted.

The secretary then read papers relating to the several sections to which they belong, after which the association adjourned to 9 o'clock this morning.

### THIRD DAY'S SESSION.

The convention was called to order at 9.30 A.M., President Mendenhall in the chair; William B. Atkinson, secretary. The reading of the minutes were, on motion, dispensed with.

Dr. Antisell then read the names of a number of gentlemen who were admitted as members on invitation.

Dr. Sayre asked that a committee be appointed to examine the charges circulated against him through the country. He requested that a special committee be appointed to report on the matter at this session.

Dr. —— said that there being a difficulty between Drs. Sayre and Ruppaner, he objected to any committee being appointed, because the other party was absent.

Dr. Murphy, of Ohio, moved that the whole matter be referred back to the society at New York.

Dr. Sayre said it was due to the association that these charges be looked into.

Dr. Murphy said that the reputation of Dr. Sayre was not damaged in this society, and he therefore insisted on his motion.

Dr. Keller, of Kentucky, reported on the part of the committee on Ethics that that committee had been forced by the press of woık to return the papers in the case of Dr. Sayre for the further consideration of the association.

Dr. Maddox moved that the whole matter be laid on the table. It was so ordered.

Dr. Yandell then moved that the delegate that had been sent as a representative to the British Medical Association be heard.

Dr. Pinkney, United States navy, representative of the American Association in England, made a long and interesting report of his visit and observations to the medical schools of Britain. The report was listened to with much attention throughout.

A vote of thanks was tendered Dr. Pinkney, and the report referred to the Committee on Publication.

Dr. F. G. Smith, of Pennsylvania, chairman of the Committee on Nomenclature, submitted a report of the names of diseases, accompanied by a resolution recommending the adoption of the nomenclature of diseases prepared by the Royal College of Physicians at London.

Dr. Underhill, of New York, also read a paper on the same subject, which was laid on the table.

The resolution recommended by the committee was discussed at some length by Drs. McDaniels, of Alabama, Logan of Louisiana, and others, who all held that revision of the nosological tables now in use was imperative.

The report, with resolution as recommended, was adopted.

Dr. C. C. Cox offered a resolution, which was adopted, for the appointment of a special committee to wait upon the Surgeon-General of the United States, and to request the privilege of duplicating the photo-microscopic slides of the tissues, so admirably executed by the indefatigable industry and skill of Surgeon J. J. Woodward, to be prepared under the direction of said committee, and distributed at a fair price to such medical colleges and institutions as may desire their use.

Dr. Bemiss, of Louisiana, from the Committee on Nominations, reported the additional standing committees for the ensuing year, which report was adopted.

Dr. Antisell offered a resolution of thanks to the Surgeon General of the United States army, for the beautiful and instructive exhibition of last evening, and recommending Dr. Woodward and Dr. Otis to the consideration of the Secretary of War, as worthy of promotion for their efforts to advance medical education in the military service.

Dr. C. C. Cox then offered a resolution of condolence with the family of the late Alden March, of New York, and that a copy of the same be sent to Dr. Alden March's bereaved family. The resolution was concurred in.

Dr. Steine, of New York, offered a resolution that the State and county authorities be requested to aid in the support of veterinary colleges in each of the States by appropriations or otherwise. Adopted.

Also, that one or more veterinary surgeons be associated with other physicians in the boards of health when they are appointed by the Governors. Lost.

Also, that veterinary surgeons be appointed to the army, with the rank of full surgeons, and also in the Agricultural Department.

Dr. Otis moved as a substitute, that the first clause relating to appointments of veterinary surgeons to the United States army be stricken out, and that the Government appoint a veterinary surgeon to the Agricultural Department with a suitable salary. Adopted.

The hour for special business having arrived—

Dr. Storer, of Boston, moved, upon behalf of the Gynæcological Society of that city, that the action of the association in 1869, condemnatory of cards by specialists in journals of a strictly medical character, should be rescinded upon the ground of abstract right and long custom with reference to the insertion of such cards. Tabled.

A resolution was offered that a committee of three be appointed to wait upon Congress and request them to regulate the quarantine laws. Adopted.

The report from the delegate to the Canadian Medical Association was received and referred to the Committee on Publication.

The report of the Committee on Communications was adopted, and the committee continued.

Dr. Stewart, District of Columbia, offered a resolution that gentlemen not members of the association were not eligible to serve on its committees. Tabled.

A resolution was offered regulating the duties of superintendents of hospitals. Adopted.

A resolution was offered that as certain so-called medical works had been published which were injurious to the reputation of the profession:

*Resolved,* That any person signing his name as author of such work shall be refused membership in this association. Adopted.

Dr. Jones, of the District of Columbia, submitted a tabular statement relating to the medical institutions and colleges throughout the country, embracing much valuable and interesting information, which after being read was referred for publication.

It was resolved that at the future meetings of the association a dinner should be given on the third day of the convention, at the expense of the members eating the dinner.

Dr. Mussey, of Ohio, offered the following:

*Resolved*, That that clause in the by-laws which provides that every alternate meeting of the association be held at Washington be repealed, and that in the future the place of meeting should be determined at each session of the association.

The resolution was concurred in.

Dr. Kerwin, of Pennsylvania, then read a lengthy report on the treatment of the insane, which was received and referred to the Committee on Publications.

Dr. White, of Buffalo, offered a resolution that the different medical schools of the country establish chairs on mental disorders. Adopted.

The Committee on Prize Essays submitted a report, which was adopted.

A resolution was offered that a committee be appointed to report what, if any, legislative means could be taken to prevent the spread of epidemic diseases. Adopted.

Dr. C. C. Cox inquired if the Committee on Ethics would make any further report of the weighty matters before them.

Dr. Antisell called attention to a paragraph from the *Chronicle* of yesterday morning, charging the Committee of Arrangements with certain actions that should be denounced by the association. He denied the charges and asked the association to sustain the committee in its actions.

On motion, it was decided to postpone the further consideration of the subject until after the report of the Committee on Ethics had been received.

Dr. N. S. Davis, of Illinois, then submitted, on behalf of the majority of the Committee on Ethics, the following

REPORT.

It appears that the matters reported to your Committee of Registration, and so much of the action of the majority of same committee as relates to the same subjects, embraces the three following subjects:

First. A charge that the majority of the Registration Committee had refused to register the delegates presenting credentials from several societies, colleges and hospitals, in the District of Columbia which claimed the right to representation.

Second. Direct charges against the Washington Society and the Medical Association of the District of Columbia, accompanied by a protest against the admission of delegates for those bodies,

Third. Direct charges, which had been lodged with the Committee of Registration, against the National Medical Association of the District of Columbia, accompanied by a protest against the registration of delegates from that society and from such other institutions as were supplied with medical officers who were members of that society.

In regard to the first charge, your committee find, on investigation, that the Registration Committee have duly registered all the delegates from all the medical institutions claiming representation in the District of Columbia, in accordance with the usages and by-laws of the association, except the Medical Society of the Alumni, of Georgetown College, the National Medical Society, the Howard Medical College, the Freedmen's Hospital, and the Small-pox Hospital, these being the institutions included in the charges already mentioned in the third specification.

It remains, therefore, only to consider the second and third specifications, and your committee ask leave to report on these separately. In relation to the second we unanimously recommend the following resolution :

*Resolved,* That the charges offered by Dr. Reyburn, as a minority of the Committee on Registration, against the Medical Society and the Medical Association of the District of Columbia, are not of a nature to require the action of the American Medical Association, the first charge referring to a duty imposed on the Society by Act of Congress, and the second referring to a matter which does not come in conflict with any part of the code of ethics.

*Resolved,* That so far as relates to the Medical Society of the Alumni, of Georgetown College, it has been shown to us that the society has sixty resident members, and is therefore entitled to six delegates instead of as requested by the committee.

In regard to the third proposition relating to the National Medical Society, Howard University Medical College, the Freedmen's Hospital, and the Small-pox Hospital, we recommend the following :—

*Resolved,* That the duties of the Committee of Arrangements, so far as relates to the registration of members, is purely clerical, consisting in the verification of the certificates of delegates and a report on the same. If credentials in proper form are presented from any society or institution professing such few as would place it *prima facie* in the list of institutions enumerated in the constitution of the association, as entitled to representation, but against which charges have been made or protests presented, the names of the delegates presenting such credentials, together with the charges or protests in the possession of the committee, should be represented to the association for its action.

*Resolved,* That the charges lodged with the Committee of Arrangements, against the eligibility of the National Medical Society of the District of Columbia, have been so far sustained that we recommend that no member of the society should be received as delegates at the present meeting of this association.

N. S. DAVIS,
H. F. ASKEW,
J. M. KELLER,

Dr. Alfred Stille, of Pennsylvania, then submitted the following as a

MINORITY REPORT.

The undersigned, members of the Committee on Ethics, while subscribing to the greater portion of the report of the majority, feel it their duty, nevertheless, to dissent from the final resolution recommending the exclusion of the members of the National Medical Society of the District of Columbia from the present meeting of this association; they offer, therefore, in lieu of that resolution the following:

*Whereas,* The institutions excluded from representation by the action of the Committee on Credentials, viz.: The National Medical Society, the Howard Medical College, the Freedmen's Hospital, and the Small-pox Hospital, are regularly organized as the constitution of the association requires; and whereas the physicians so excluded are qualified practitioners of medicine, who have complied with all the conditions of membership imposed by the association; and whereas, in the judgment of the undersigned no sufficient ground exists for the exclusion of such institutions and physicians from this association; therefore

*Resolved,* That the institutions above named are entitled to representation, and that the physicians claiming to represent them are entitled to seats in the American Medical Association.

ALFRED STILLE,
J. S. WOODWARD.

Motions were made to accept and reject the different reports, when, amidst the greatest excitement, the yeas and nays were called for.

Dr. Howard, of the District of Columbia, asked who of the District were entitled to vote.

The Chair then decided that those gentlemen were entitled to vote who had been unanimously admitted by the Committee on Ethics.

Dr. Cox endeavored to speak, but, amid cries of "Sit down," was forced to desist.

An appeal was taken from the decision of the Chair, which was not sustained, the vote being 115 for and 90 against.

The secretary began to call the roll upon the question of laying the minority report upon the table about 1.20 and continued until 2 o'clock, when the secretary announced the vote—yeas 107, nays 85. The minority report was accordingly tabled.

The greatest excitement prevailed throughout the calling of the names.

A motion was made to adopt the majority report.

Dr. C. C. Cox, of Maryland, then addressed the association, protesting against its action in rejecting the minority report, and gave a brief history of the origin of the differences of opinion now existing among the several societies of the city. Dr. Cox during his address was frequently called to order.

The question on the adoption of the majority report was then called, but it was thought to be unnecessary, as the rejection of the minority report adopted it.

RECEPTION AT MAYOR BOWEN'S.

Last evening the Medical Association visited the Capitol for the purpose of seeing the dome lighted, with which operation they all expressed themselves much gratified. The association, *en masse*, then called upon Mayor Bowen, filling the house to such an extent that anything more than a formal reception was impossible. The evening, however, passed off very pleasantly.

### FOURTH DAY'S SESSION.

The association assembled in the morning at Lincoln Hall, Professor George Mendenhall in the chair, and Professor W. B. Atkinson, secretary.

A resolution was offered by Dr. J. J. Woodward, United States army, that the Surgeon General be requested to authorize Dr. Woodward to make copies of his photo-microscopic slides, to be distributed at a fair price to such medical colleges and institutions as may desire their use. Adopted.

A debate then occurred on the duties of the Committee on Ethics in the matter of passing on the credentials of delegates representing institutions which admit women to the practice of medicine.

Professors Hartshorn, Bell, Davis, Maddox, and Cohen, participated in the debate, after which the matter was indefinitely postponed.

Dr. Palmer, of Maine, offered a resolution of inquiry as to why the Howard Medical College had been excluded from admission in this association, stating that the institution had been chartered by special act of Congress, and was recognized all over the country as a first-class college.

A discussion took place on the adoption of the resolution.

Dr. N. S. Davis, of Illinois, said if the resolution was withdrawn he, as chairman of the Committee on Ethics, would give his reasons in writing why the institution was excluded.

The resolution was withdrawn.

Dr. R. J. O'Sullivan, of New York, then offered the following:

*Whereas,* Apothecaries are accustomed to renew medicines prescribed by physicians without due authority from the physician, thereby doing much injury to patients, and by which many lives have been destroyed; and as apothecaries are unwilling to discontinue the practice except by a general action—therefore,

*Resolved,* That this association take such action as will bring about the discontinuance of the practice.

Referred to a special committee.

A protest against the adoption of Dr. Pinkney's report of the medical corps of the navy was submitted, and after some debate was laid on the table, and the whole subject referred to a committee of three, to report at the next meeting of the association.

The Committee on Ethics made reports on several cases relating to charges against individuals and colleges in the different States, and they were referred to appropriate committees.

A paper was read on the treatment of the insane; which was referred to the Committee on Publication.

Dr. Hartshorn, of Philadelphia, offered a resolution that the constitution be so amended as not to exclude women from membership of this association. Laid on the table.

Dr. Powell, of Atlanta, Ga., offered a resolution that the association do not recognize any college or institution against which charges are pending.

It was opposed by Dr. Reyburn, of the District of Columbia, and advocated by Dr. Powell, after which it was laid over under the rules.

Dr. Lee offered a paper on insane institutions; which was referred to the Committee on Publication.

A paper on epidemic diseases was read and referred.

A vote of thanks was tendered to Mayor Bowen, for entertaining the association at his residence on Wednesday night.

A motion was made that the next meeting of the association be held in San Francisco, California, in June next.

A resolution declaring Dr. Horace Wells, of Boston, to be the discoverer of anæsthesia, was adopted.

A resolution of thanks was tendered to Dr. Mendenhall, for the able manner in which he has presided over the deliberations of the association.

Dr. John Sullivan, of Massachusetts, offered the following:

*Resolved,* That no distinction of race or color, shall exclude persons claiming admission to this association who are duly accredited thereto.

During its reading the speaker was met with a storm of hisses, which compelled him to stop. Cries of "go on," "go on," were heard, and he said he would do so when the serpents became quiet. He then finished its reading, and was allowed to speak seven minutes. He spoke as follows:

### SPEECH OF DR. JOHN SULLIVAN.

MR. PRESIDENT AND GENTLEMEN OF THE AMERICAN MEDICAL ASSOCIATION,—I rise to offer a resolution, which I venture to affirm is one of the most important which has ever been presented for the consideration of this body. The importance of this resolution to an American national medical association can hardly be exaggerated inasmuch, as its passage or the reverse involves many of the highest questions in social science and political economy. Eminently proper it is that this resolution should receive due consideration from the representatives of so many of the scientific bodies of a country whose territorial limits embrace half a continent, and every variety of climate, soil, and race; a country where, by the fusion of so many different nationalities, a homogeneous race is in gradual process of formation, and an entirely new civilization is being step by step evolved. Gentlemen, the resolution is this: "*Resolved*, That no distinction of race or color shall exclude from this organization any person duly accredited thereto." Gentlemen, in moving this resolution I present in its abstract form the question which in reality underlies the controversy we are this day called to decide. This is a scientific body, gathered from every section of the country. We have met to deliberate on purely scientific subjects; to determine questions of purely scientific character—questions which concern not our own welfare, but the welfare and perpetuity even of mankind. I will not, however, deny that, in part, at least, this association was organized for social purposes, but its main objects are those which I have indicated. Now, is there one gentleman present from the North, South, East or West, who would hesitate to admit to this floor the duly accredited representative of any scientific body, organized by any race under the sun, no matter what might be the color of his skin, provided it were not black?

Why is it, gentlemen, that you ostracise no qualified persons save persons of this one color? Why do you entertain an inveterate hostility to the Ethiopian being represented here, and yet as we all believe, make no objection whatever to those whose complexion betray their origin from some one of the other four great divisions of the human family? For my part I am satisfied that our friends from the South greatly mistake the nature of their feelings toward the negro. There is a mass of living evidence which renders irresistable the conclusion that however much they may dislike the black man, toward the black woman they entertain, many of them, the liveliest feelings of our nature. But to return; comparatively speaking, there is nothing in our present state of existence of real value save ideas. The mind, meaning therewith to include not only the intellect but the affections, is that which constitutes the man, the man made in the image of his Maker, endowed with god-like attributes of intelligence and destined for an endless existence. All the other attributes of humanity are but mere accessories of the mind, all else hastens rapidly to decay—"Shadows are we, what shadows we pursue." The question before us, then, is purely of qualification, of capacity; it is a question of education, a question of intelligence, intellect, in short, gentlemen, of brains, *brains, brains, brains!* Intellect is at once the evidence and the seal of manhood.

> "The *skin* is but the guinea's stamp;
> A man's a man for a' that."

For my own part, I would not refuse to admit to the deliberations of this great scientific body, provided he came duly accredited thereto, an ourang outang with a tail ten feet long; and could he show me an interesting case of disease of that tail, I should be very ready to examine that case with him, and to receive all the light which his experience could shed upon it. I would say to him "God speed" in his effort to heal on scientific principles the diseases ot his brother babboons. But I am told, gentlemen, and you may be told, by our brethren from the South, that it is the social element of this organization which lies at the bottom of their opposition to the admission of the African to this floor. As one of them has said to me, if Julius Augustus Cæsar presents his credentials and becomes entitled to a seat here, we must meet him, not only on this floor, but elsewhere—at the Mayor's reception, or at any other place where we meet as gentlemen for recreation or for social intercourse with each other. He may bring with him his wife and children, and we may bring with us our wives and children, and *they* will have to meet on terms of social equality, Mrs. Cæsar and all the little Cæsars. And this, say gentlemen from the South, is a bitter pill to swallow. It *is* a bitter pill to swallow, I admit; but is it any worse, any harder, any more humiliating, any more cruel for *you* than it is for *us?* Are not we, too, Caucasians? Do we ask you to taste the cup which we are not ourselves forced to drain? Who and what are *you* more than *we?* There is as good blood in New England as there is in the South, in Massachusetts as there is in Kentucky. Two hundred and thirteen years ago, this year, if I rightly remember the date when he landed on these shores my original ancestor in this country first planted his feet on New England soil. Who is he? (pointing to Dr. Yandell of Kentucky.) Gentlemen from the South, in God's name who are *you* who dare to affirm that to meet the African on terms of social equality is a harder thing, a more humiliating thing for *you* than it is for *us?* No, gentlemen, the dose we compel you to take is one which we must ourselves swallow. And now, gentlemen, inasmuch as the political status of the African is now one with yours and mine; now that he is admitted to the Senate chamber of the United States; now that he may stand there at least on terms of social equality, and in the presence of the Chief Magistrate of this country, let not this great scientific body, embracing as it does, with but few exceptions, the wisest men in our profession; I repeat let not this great scientific body prove itself unworthy of the high trusts confided to its charge. Let us not deny to the enfranchised African, the same right in the Republic of letters which are now fully and cheerfully accorded to him in the political organization under which we live. Let us not deprive him of the opportunity which the sword has opened for him of proving to the civilized world, if he can do this, that inasmuch as he displays the essential qualities of manhood, he has a right to be accounted and treated as a man.

During the delivery of the above speech great confusion reigned, and had it not been for the persistent efforts of Dr. Yandell, of Louisville, Ky., at one time surgeon-in-chief of Kirby Smith's army, Dr. S. would not have been able to have concluded his remarks. During their delivery Dr. Yandell appealed to the sense of the convention to allow him to proceed, stating that he was a Southern representative, but that he desired fair play, and trusted that Dr. Sullivan would be heard.

Upon the conclusion of Dr. Sullivan's remarks, Dr. N. S. Davis, of Chicago, read the following

### REPORT OF THE COMMITTEE ON ETHICS.

In reply to the resolution of the association calling upon the majority of the Committee on Ethics for the reason why they in their report exclude the delegates from the Medical Department of Howard University, they respectfully state that there is nothing in the report which directly excludes delegates from the said University or any other medical institution in the District of Columbia, except the National Medical Society.

The resolution on this subject reported by the committee, is in these words:

*Resolved*, That the charges lodged with the Committee of Arrangements against the eligibility of the National Medical Society of the District of Columbia, have been so far sustained that we recommend that no members of that society should be received as delegates at the present meeting of the association.

It will be seen that the only parties excluded from admission as delegates at the present meeting are the members of the National Medical Society. If the Medical Department of Howard University had chosen to send any delegates who are not members of that society there is nothing whatever in the report to prevent them from being received.

In the papers referred to your Committee on Ethics, were a list of charges with specification in the usual form against the registration of the National Medical Society. These charges may be clearly stated as follows:

1. That said National Medical Society recognizes and receives as members medical men who are not licentiates, and who are acting in open violation of sections 3, 4 and 5, of the law of Congress constituting the charter of the Medical Society of the District of Columbia.

2. That a large part of the members of the National Medical Society are also members of the National Medical Association of the District of Columbia, and are openly and freely violating the rules and ethics of the association to which they have subscribed.

3. That they have both in its capacity as a society and by its individual members misrepresented the action of the Medical Society and the Medical Association of the District of Columbia, and used unfair and dishonorable means to procure the destruction of the same, by inducing Congress to abrogate their charter.

Each and all of these charges were, in the opinion of the majority of the committee, fully proved by the members of the National Medical Society themselves, who appeared voluntarily before your committee as witnesses. Therefore, if we have any regard for the maintenance of the laws of the land on the ethics of our medical organization, the undersigned could not come to any other conclusion than was expressed in the last resolution recommended by the majority of the Committee on Ethics.

Dr. Robert Reyburn rose to reply to the report, but was called to order, as not being a member. Finally the Convention allowed him five minutes to speak.

Dr. Reyburn said that he had never violated the code of ethics, and had favored the admission of colored men to the college he belonged. No man should be excluded on account of color. His resolution had not been received by the old society, and he was now a member of the National Medical Society. If that constituted a violation of the code of ethics he plead guilty. Last year he had been chairman of the Committee on Credentials at New Orleans, and when about to make his report he was shamefully treated by the Committee of Arrangements.

Dr. Antisell denied the assertion.

Dr. Loomis, of the District of Columbia, stated that he was a member of the Faculty of Medicine of Howard University, and he could see no reason why he was excluded. He then offered a resolution to the effect that the members of the Committee on Ethics who signed the majority report be censured for so doing.

His resolution was laid on the table.

Dr. Johnson, of the District of Columbia, President of the Medical Society of the District of Columbia, then proceeded to give a detailed history of the difficulties existing in our local societies. He also stated that Dr. D. W. Bliss had violated the rules of ethics by having his name printed on a bill of fare at Willard's Hotel.

Dr. Bliss denied its having been placed there with his knowledge.

Drs. Johnson, Busey and Marbury sustained the charge by statements.

After which Dr. Busey replied to certain statements of Dr. Johnson, and read the sixteenth rule of the Code of Ethics, showing that the code had been violated in the attempt to force the colored man upon the society. This was what had caused all the trouble. Dr. Borrows had been instrumental in bringing about the color difficulty. He denied that politics was the cause of the difficulty, as had been stated by Dr. Cox.

The vote was then taken on Dr. Sullivan's resolution, and it was tabled by a vote of 106 yeas to 60 nays.

Dr. H. R. Storer, of Boston, offered the following:

That inasmuch as it has been distinctly stated and proved, that the consideration of race and color has had nothing whatever to do with the decision of the question of the reception of the Washington delegates, and inasmuch as charges have been made in open session to-day distinctly attaching the stigma of dishonor to parties implicated, which charges have not been even denied by them, though present; therefore.

*Resolved,* That the report of the majority of the Committee of Ethics be declared as to all intents and purposes unanimously adopted by the association.

The resolution was adopted by a vote of 112 yeas to 37 nays.

The following was the report of the Committee on Ethics, to which was referred the protest by Drs. Storer and Sullivan, on behalf of the Gynæcological Society of Boston, against the representation of the Massachusetts Medical Society.

"Whereas the charge of tolerating in the Massachusetts Medical Society men acknowledged to have become homœopaths and eclectics is fully proved, and is plainly a violation of the Code of Ethics; but, inasmuch, as it also appears, that the parties making the charges here, being themselves members of the same Society, have not previously made or caused to be made, any specific charges against such irregular practitioners in the proper form before the Massachusetts Medical Society or its councillors; it is the opinion of the Committee that such steps should have been taken and the results obtained before appealing to this Association, and

It is therefore recommended: that the Committee of Registration should register all regularly accredited delegates from the Society to the present meeting. This Committee further recommend that, unless said Society takes the necessary steps to purge itself of irregular practitioners, it ought not to be entitled to further representation in this Association.

The association then adjourned *sine die.*

